# Facilitated Event-Related Power Modulations during Transcranial Alternating Current Stimulation (tACS) Revealed by Concurrent tACS-MEG

**DOI:** 10.1523/ENEURO.0069-18.2018

**Published:** 2018-07-25

**Authors:** Florian H. Kasten, Burkhard Maess, Christoph S. Herrmann

**Affiliations:** 1 Experimental Psychology Lab, Department of Psychology, European Medical School, Cluster for Excellence “Hearing for All”, Carl von Ossietzky University, Oldenburg, Germany; 2 Neuroimaging Unit, European Medical School, Carl von Ossietzky University, Oldenburg, Germany; 3 MEG and Cortical Networks Group, Max Planck Institute for Human Cognitive and Brain Sciences, Leipzig, Germany; 4 Research Center Neurosensory Science, Carl von Ossietzky University, Oldenburg, Germany

**Keywords:** Cognitive performance, event-related oscillations, MEG, online effects, transcranial alternating current stimulation (tACS)

## Abstract

Non-invasive approaches to modulate oscillatory activity in the brain are increasingly popular in the scientific community. Transcranial alternating current stimulation (tACS) has been shown to modulate neural oscillations in a frequency-specific manner. However, due to a massive stimulation artifact at the targeted frequency, little is known about effects of tACS during stimulation. It remains unclear how the continuous application of tACS affects event-related oscillations during cognitive tasks. Depending on whether tACS influences pre- or post-stimulus oscillations, or both, the endogenous, event-related oscillatory dynamics could be pushed in various directions or not at all. A better understanding of these effects is crucial to plan, predict, and understand outcomes of solely behavioral tACS experiments. In the present study, a recently proposed procedure to suppress tACS artifacts by projecting MEG data into source-space using spatial filtering was utilized to recover event-related power modulations in the alpha-band during a mental rotation task. MEG data of 25 human subjects was continuously recorded. After 10-minute baseline measurement, participants received either 20 minutes of tACS at their individual alpha frequency or sham stimulation. Another 40 minutes of MEG data were acquired thereafter. Data were projected into source-space and carefully examined for residual artifacts. Results revealed strong facilitation of event-related power modulations in the alpha-band during tACS application. These results provide first direct evidence that tACS does not counteract top-down suppression of intrinsic oscillations, but rather enhances pre-existent power modulations within the range of the individual alpha (= stimulation) frequency.

## Visual Abstract

**Figure:**
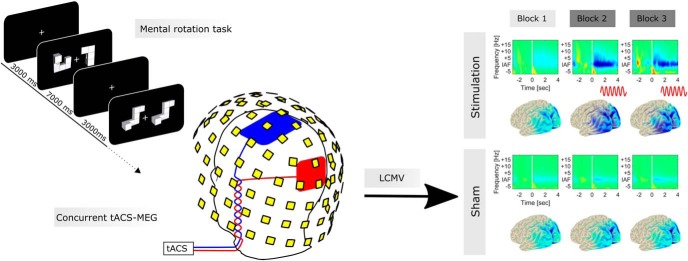

## Significance Statement

Transcranial alternating current stimulation (tACS) is increasingly used in cognitive neuroscience to study the causal role of brain oscillations for cognition. However, online effects of tACS largely remain a “black box” because of an intense electromagnetic artifact encountered during stimulation. The current study is the first to employ a spatial filtering approach to recover, and systematically study, event-related oscillatory dynamics during tACS, which could potentially be altered in various directions. TACS facilitated pre-existing patterns of oscillatory dynamics during the employed mental rotation task, but did not counteract or overwrite them. In addition, control analyses and a measure to quantify tACS artifact suppression are provided that can enrich future studies investigating tACS online effects.

## Introduction

Oscillatory activity of neuronal assemblies is a ubiquitous phenomenon in the brain observed within and between different brain structures and across species (Buzsáki, 2006). Over the past decades, these oscillations have been linked to a variety of brain functions, such as memory, perception, and cognitive performance (Klimesch, 1999, 2007; Bașar et al., 2000; Buzsáki, 2006). Traditionally, these relationships were fruitfully investigated using imaging techniques such as electro- or magnetoencephalography (EEG/MEG). However, in their nature, these approaches are correlational and cannot resolve causal relationships between neural oscillations and cognitive processes. The recent (re-)discovery of non-invasive transcranial electrical stimulation (tES) now allows to directly probe these causal relationships (Herrmann et al., 2016b).

The application of oscillatory currents through the scalp by means of transcranial alternating current stimulation (tACS) has been shown to modulate endogenous brain oscillations in a frequency-specific manner (Fröhlich and McCormick, 2010; Ozen et al., 2010; Zaehle et al., 2010; Helfrich et al., 2014). Effects of tACS during stimulation have been primarily investigated in animals (Fröhlich and McCormick, 2010; Ozen et al., 2010; Kar et al., 2017) and with computational models (Fröhlich and McCormick, 2010; Reato et al., 2010; Ali et al., 2013; Negahbani et al., 2018). Due to a massive artifact introduced to electrophysiological signals, studies on tACS effects in humans have mostly been restricted to behavioral measures (Marshall et al., 2006; Kar and Krekelberg, 2014; Lustenberger et al., 2015), blood oxygen level–dependent (BOLD)-signal effects (Alekseichuk et al., 2016; Cabral-Calderin et al., 2016; Vosskuhl et al., 2016; Violante et al., 2017), and aftereffects in M/EEG (Zaehle et al., 2010; Wach et al., 2013; Neuling et al., 2015; Veniero et al., 2015; Vossen et al., 2015; Kasten et al., 2016; Stecher et al., 2017). In case of M/EEG, a frequency specific increase in oscillatory power after stimulation is consistently reported (Zaehle et al., 2010; Neuling et al., 2013; Vossen et al., 2015; Kasten et al., 2016). It is often assumed that the underlying mechanism of action of tACS is entrainment of neural activity to the external driving force, which is observed in computational and animal models (Fröhlich and McCormick, 2010; Ozen et al., 2010; Reato et al., 2010; Ali et al., 2013; Negahbani et al., 2018). Direct evidence for entrainment of brain oscillations to tACS in humans is, however, largely missing so far.

Besides sustained effects on the power of spontaneous oscillations after the stimulation, tACS has more recently been demonstrated to alter event-related oscillatory dynamics in the context of a cognitive task (Kasten and Herrmann, 2017). In that study, event-related desynchronization (ERD) was enhanced after tACS application, accompanied by improved performance in a classic mental rotation (MR) task (Shepard and Metzler, 1971; Kasten and Herrmann, 2017). The amount of ERD in the alpha-band has previously been linked to MR performance (Michel et al., 1994; Klimesch et al., 2003). Although an increase in task performance has already been observed during tACS, the precise oscillatory dynamics during tACS remain unclear (Kasten and Herrmann, 2017). Given that many tACS studies rely solely on behavioral measures, an understanding of the effect of tACS on event-related oscillations is crucial. Depending on whether the stimulation merely affects pre- or post-stimulus oscillations or both, tACS may increase, decrease, or not modulate ERD/ERS. Each of these scenarios would result in different behavioral outcomes to be expected. The current study aims to provide a first step toward understanding the effects of tACS on event-related power modulations during stimulation. To this end, the experiment of Kasten and Herrmann (2017) was repeated in an MEG scanner. The application of linearly constrained minimum variance beamforming (LCMV; Van Veen et al., 1997) on MEG recordings has been shown to substantially suppress electromagnetic artifacts encountered during tES (Soekadar et al., 2013; Neuling et al., 2015). Although this approach will never completely remove artifacts from the signal (Noury et al., 2016; Mäkelä et al., 2017; Noury and Siegel, 2017), artifact suppression may still be sufficient to recover changes in event-related dynamics during tACS (Neuling et al., 2017; Noury and Siegel, 2018).

In the present study, LCMV was used to attempt to recover the event-related power modulations in the alpha-band encountered during MR. Based on previous behavioral results, an increase in alpha-power modulation during tACS was hypothesized (Kasten and Herrmann, 2017). The measure to capture tACS effects (absolute power difference instead of relative change) was carefully chosen to be robust against the possible influence of residual artifacts. Careful control analyses were conducted to rule out that the observed effects can be attributed to a residual artifact.

## Materials and Methods

### Participants

Twenty-five healthy volunteers were randomly assigned to one of two experimental conditions. They received either 20 min of tACS or sham stimulation during the course of the experiment. All were right-handed according to the Edinburgh Handedness Inventory (Oldfield, 1971) and had normal or corrected-to-normal vision. Participants gave written informed consent before the experiment and reported no history of neurologic or psychiatric conditions. The experiment was approved by the Commission for Research Impact Assessment and Ethics at the University of Oldenburg and was conducted in accordance with the Declaration of Helsinki. Three subjects exhibited low tolerance to skin or phosphene sensations while determining the individual stimulation intensity (see Electrical stimulation). Due to the resulting low stimulation currents (below 0.4 mA), these subjects were excluded from the analysis. Furthermore, two participants were excluded as they did not exhibit alpha modulation in response to the cognitive task during the baseline block. Data of 20 subjects (10 in stimulation group, 10 in sham group, age: 26 ± 3 years, 8 females) remained for analysis. Although the groups were initially counterbalanced for participants’ sex, the exclusion of subjects resulted in an imbalance in the sham group (7 males and 3 females vs. 5 males and 5 females in the stimulation group).

### Magnetoencephalogram

Neuromagnetic activity was recorded at a rate of 1 kHz using a 306-channel whole-head MEG system (Elekta Neuromag Vectorview, Elekta Oy) with 102 magnetometers and 204 orthogonal, planar gradiometers, sampling from 102 distinct sensor locations. An online bandpass filter between 0.1 and 330 Hz was applied. The experiment was conducted in a dimly lit, magnetically shielded room (MSR; Vacuumschmelze) with participants seated below the MEG helmet in upright position. Before the experiment, three anatomic landmarks (nasion and left and right posterior tip of tragi) were digitized using a Fastrack (Polhemus), along with the location of five head position indicator (HPI) coils, and >200 head shape samples to allow continuous head-position tracking and later coregistration with anatomic MRIs.

**Figure 1.**
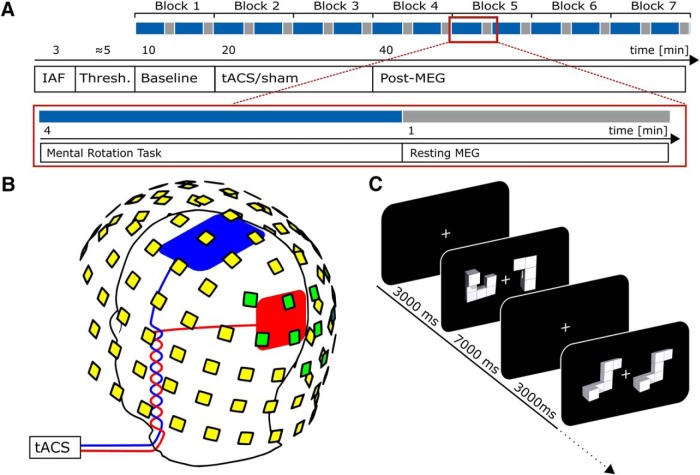
Experimental procedures. ***A***, Time course of the experiment. Blue indicates periods during which the MR task was performed; gray indicates intermittent resting periods. ***B***, Positions of stimulation electrodes (red/blue) and layout of MEG sensors (yellow/green). Stimulation electrodes were placed centered above Cz (7 × 5 cm) and Oz (4 × 4 cm) of the international 10-10 system. MEG was recorded from 102 locations. Each location contains a sensor triplet of one magnetometer and two orthogonal planar gradiometers, resulting in a total of 306 channels. Sensor locations used to determine participants’ individual alpha frequency are marked green. ***C***, Mental rotation task. Each trial started with the presentation of a white fixation cross at the center of the screen. After 3000 ms, a mental rotation stimulus (two objects) was presented and remained on screen for another 7000 ms. During this time participants were required to judge whether the two objects presented were either different (example depicted in 2nd display) or identical (but rotated; 4th display). ***A*** and ***C*** are adapted from Kasten and Herrmann (2017).

After finishing the preparations, individual alpha frequency (IAF) was determined from a 3 min, eyes-open, resting-state MEG recording. Data were segmented into 1 s epochs. Fast Fourier transforms (FFTs) were computed for each of the segments using the Fieldtrip toolbox (Oostenveld et al., 2011). The power peak in the averaged spectra, in the 8–12 Hz band, was determined in a set of posterior sensors showing most pronounced alpha activity by visual inspection. The identified frequency was used as stimulation frequency for the subsequent procedures (refer to Fig. 1*A* for an overview of the time course of the experiment and Fig. 1*B* for an illustration of sensor locations used to determine participants’ IAF).

### Electrical stimulation

Participants received either 20 min of tACS (including 10 s fade-in and fade-out) or sham stimulation (30 s stimulation in the beginning of the stimulation period, including 10 s fade-in and out) at their individual alpha frequency (IAF). The sinusoidal stimulation signal was digitally generated at a sampling rate of 10 kHz in Matlab 2012a (32-bit, The MathWorks) and transferred to a digital-analog converter (Ni USB 6221, National Instruments). From there, the signal was streamed to the remote input of a battery-driven constant current stimulator (DC Stimulator Plus, Neuroconn), which was placed inside an electrically shielded cabinet outside the MSR. The signal was then gated into the MSR via a tube in the wall using the MRI extension-kit of the stimulator (Neuroconn). Electrical stimulation was administered by two surface conductive rubber electrodes attached to participants’ scalps over electrode positions Cz (5 × 7 cm) and Oz (4 × 4 cm) of the international 10-10 system (Fig. 1*B*), using an adhesive, electrically conductive paste (ten20 Conductive Paste, Weaver and Co.). Impedance was kept below 20 kΩ (including two 5-kΩ resistors in the cables of the MRI extension-kit of the stimulator). Accordingly, impedance between the electrodes was limited to 10 kΩ.

To minimize confounding influences from either phosphene or skin sensations, tACS was applied below participants’ individual sensation threshold, using an established thresholding procedure (Neuling et al., 2013, 2015; Kasten et al., 2016; Kasten and Herrmann, 2017). To this end, participants were stimulated with an initial intensity of 500 µA at their IAF. Depending on whether participants noticed the initial stimulation, intensity was either increased or decreased in steps of 100 µA until they noticed/not noticed the stimulation. The highest intensity at which participants did not notice the stimulation was subsequently used as tACS intensity in the main experiment. The thresholding was performed for both groups to keep experimental procedures similar. The obtained intensities for the sham group were applied during the 30 s stimulation train in the beginning of the stimulation block (see above). Three participants exhibited sensation thresholds below 400 µA and were excluded from analysis. On average, participants were stimulated with 715 µA ± 301 µA (peak-to-peak; stimulation group: 680 µA ± 175 µA) at a frequency of 10.5 Hz ± 0.9 Hz. TACS or sham stimulation was applied, immediately following the baseline block, for 20 min during the second and third blocks of the behavioral experiment.

### Mental rotation task

Visual stimuli were presented using Psychtoolbox 3 (Kleiner et al., 2007) implemented in the same Matlab code that generated the electrical stimulation signal. Visual stimuli were rear-projected onto a screen inside the MSR at a distance of ∼100 cm from the participant.

Subjects performed the same MR paradigm that was employed in a recent tACS-EEG study (Kasten and Herrmann, 2017). Stimuli were taken from an open-source stimulus set (Ganis and Kievit, 2015), comprising 384 MR stimuli (pairs of 2-dimensional objects) similar to the objects used in the seminal paper of Shepard and Metzler (1971). The duration of the experiment was reduced from 8 to 7 blocks of 10 min each. Participants were familiarized with the task on a laptop during electrode preparation (16 practice trials with immediate feedback). All other parameters were kept similar. Each block consisted of 48 trials, starting with the presentation of a white fixation cross at the center of the screen. After 3000 ms, an MR stimulus was presented for 7000 ms. During this time, participants were asked to judge whether the two objects on the screen were either identical (can be brought into alignment by rotating) or different (cannot be brought into alignment by rotating) by pressing a button with their left or right index finger (Fig. 1*C*). To keep visual stimulation at a constant level, the MR stimuli remained on screen for the whole 7000 ms, regardless of participants’ reaction times. Every 24 trials, the task was interrupted by a 1 min resting period during which a rotation of the fixation cross had to be detected. This ensured that participants remained focused and tried to avoid head movements. The first block served as a baseline measurement before stimulation. During the second and third block, tACS or sham stimulation was applied. The remaining four blocks served as post-stimulation measurements to capture aftereffects of the stimulation (Fig. 1*A*). The experiment had a total duration of 70 min.

### Debriefing

After finishing the experiment, participants filled out a translated version of a questionnaire assessing commonly reported side effects of transcranial electrical stimulation (Brunoni et al., 2011). Subsequently, they were asked to indicate whether they believe they received tACS or sham stimulation. Finally, all subjects were informed about the aims of the experiment and their actual experimental condition.

### Data analysis

Data analysis was performed using Matlab 2016a (The MathWorks). MEG data processing was performed using the Fieldtrip toolbox (Oostenveld et al., 2011) embedded in custom Matlab scripts.

#### Behavioral data

Analysis of performance and reaction time (RT) data followed the approach of Kasten and Herrmann (2017). Performance, in percentage correct, in each block (48 trials) was calculated and normalized by pre-stimulation baseline to account for interindividual differences. The resulting values reflect performance change in each block relative to baseline. RTs were averaged separately for each rotation angle and normalized by their respective baseline RT. The normalized RTs were then averaged over angles for each block. This procedure accounts for the known increase in RT with larger rotation angles (Shepard and Metzler, 1971).

#### MEG processing and artifact suppression

MEG data were resampled to 250 Hz and filtered between 1 and 40 Hz using a fourth-order, zero-phase Butterworth filter. Data were projected into source-space by application of a linearly constrained minimum variance (LCMV) beamformer (Van Veen et al., 1997), a procedure that has been demonstrated to suppress artifacts originating from transcranial electrical stimulation (Soekadar et al., 2013; Neuling et al., 2015). Filter coefficients were individually estimated for each block using the noise covariance matrix, an equally spaced (1.5 cm) 889-point grid warped into Montreal Neurologic Institute (MNI) space, and single-shell headmodels (Nolte, 2003), created from individual T1-weighted MRIs. MRIs were coregistered to the median head position in each block, estimated from continuous HPI signals using the Elekta Neuromag MaxFilter software (Elekta Oy). The signal-space separation method (Taulu et al., 2005) offered by the software was not applied, as it seemed to corrupt tACS artifact suppression after beamforming. Covariance matrices were estimated by segmenting each MEG recording into 2 s epochs. The regularization parameter λ for the LCMV beamformer was set to zero to ensure optimal artifact suppression, as suggested by Neuling et al. (2017).

Sensor-space MEG data were segmented –5 to 7 s around the onset of the MR stimuli. Epochs were then projected into source-space using the previously obtained beamformer filters, resulting in 889 virtual channels, distributed over the brain. A time–frequency analysis was computed for all trials using Morlet wavelets with a fixed width of 7 cycles. The resulting time–frequency spectra were averaged for each block.

As mentioned above, all analysis procedures in this study were rigorously checked with respect to their robustness against the influence of residual artifacts in the data (Noury et al., 2016; Neuling et al., 2017). This involved a careful choice of the measure used to capture event-related changes in oscillatory power. Traditionally, such changes have been evaluated using the concept of event-related (de-)synchronization (ERD/ERS), which has been defined by Pfurtscheller and Lopes Da Silva (1999) as:

(1) $$ERD/ERS=\frac{R-A}{R}*100,$$

where *R* is the oscillatory power within the frequency band of interest during a reference period, before stimulus onset, and *A* is the power during a testing period after stimulus onset. However, assuming that residual tACS artifacts (*R_Res_* and *A_Res_*) are equally contributing to *R* and *A*, this would change the equation in the following way:

(2) $$ERD/ERS=\frac{\left(R+R_{\text{Res}}\right)-\left(A+A_{\text{Res}}\right)}{\left(R+R_{\text{Res}}\right)}*100.$$

Given that the residuals in *R* and *A* are uncorrelated with the task and have approximately equal strength (*R_Res_* ≈ *A_Res_*), their influence cancels out in the numerator but biases the denominator of the equation, resulting in systematic underestimations of the observed power modulations:

(3) $$ERD/ERS=\frac{R-A}{\left(R+R_{\text{Res}}\right)}*100.$$

For this reason the pure difference between reference and testing period (for the sake of clarity referred to as event-related power difference; ERΔ_Pow_) was used to more accurately capture event-related power modulations in the current study:

(4) $${ER\Delta }_{Pow}=\left(\text{R}+R_{Res}\right)-\left(A+A_{Res}\right)=R-A.$$

Power in the individual alpha-band (IAF ± 2 Hz) was extracted with the reference and test periods ranging from –2.5 to –0.5 s before and 0 to 2 s after stimulus onset, respectively.

Performance of the artifact suppression was evaluated by estimating the size of the residual artifact relative to the brain oscillation of interest (see Evaluation of artifact suppression). As will be described in more detail in Results, the beamformer successfully suppressed the tACS artifact from ∼2,500,000 times the size of human alpha oscillations down to a factor of <3. However, some “hot spots” showing larger residual artifacts (1:10) are apparent in the proximity of stimulator cables and the central stimulation electrode. To avoid the inclusion of virtual channels in the analysis that contain strong residual artifacts but no physiologically meaningful effects, brain areas showing strongest alpha-power modulation in response to the onset of the MR stimuli were localized based on the first (artifact-free) block before stimulation. To this end, a dependent-sample random permutation cluster *t*-test (two-tailed) with 5000 randomizations and Monte Carlo estimates to calculate *p*-values was run to compare power in the IAF-band between the reference and test periods during the baseline block. The test was performed on the whole sample (stimulation and sham group pooled). Clusters were thresholded at an α-level of 0.01. The resulting significant negative cluster was used as a region of interest (ROI) to extract the time course of ERΔ_Pow_ from each block. To account for interindividual differences, ERΔ_Pow_ in each block was normalized by ERΔ_Pow_ in the baseline block before stimulation. To test whether the effects of tACS were specific to the alpha-band, the same analysis was performed on power modulations in the lower (IAF + 3 Hz to IAF + 11 Hz) and upper (IAF + 12 Hz to IAF + 20 Hz) beta-bands within the ROI.

#### Evaluation of artifact suppression and control analyses

As discussed earlier, the application of LCMV beamforming results in a strong, yet imperfect, suppression of the tACS artifact (Noury et al., 2016; Mäkelä et al., 2017; Noury and Siegel, 2017). It is therefore crucial to characterize the achieved artifact suppression and rule out the possibility that the effects observed during stimulation result from residual artifacts in the data, rather than a true effect of tACS on the brain.

To evaluate the artifact suppression achieved by the spatial filtering procedure, participants’ alpha-power (IAF ± 2 Hz) was extracted from the pre-stimulus interval of the baseline and the two stimulation blocks. The power in the baseline block provides an estimate of participants’ natural, artifact-free alpha-power, which can be compared to the power encountered during stimulation blocks before (on the sensor-level) and after (on the sensor-level) beamforming. It is therefore possible to roughly estimate the size of the stimulation artifact relative to the brain signal of interest. This artifact-to-brain-signal-ratio was calculated for each magneto- and gradiometer channel as well as for each virtual channel after LCMV. While this measure is not able to disentangle brain signal/tACS effects from a residual artifact after LCMV, it can provide an upper boundary for the size of the residual artifact and allows the inspection of its spatial distribution.

A major assumption of the presented analysis framework, for event-related power modulations during tACS, is that the (residual) artifact has similar strength during the pre- and post-stimulus intervals, such that its influence cancels out when contrasting (subtracting) the two intervals (Eq. 4). Previous studies have demonstrated that physiological processes such as heartbeat and respiration can result in impedance changes of body tissue and small body movements, which change the size of the tACS artifact (Noury et al., 2016; Noury and Siegel, 2017). To rule out a similar modulation of artifact strength occurring in an event-related manner accounting for potential effects observed on the source-level, a control analysis was conducted. Sensor-level MEG time-series during the two stimulation blocks were bandpass-filtered around the stimulation frequency (IAF ± 1 Hz), and the signal envelope was extracted using a Hilbert transform. The envelope time series was subsequently segmented analogously to the ERΔ_Pow_ analysis and demeaned. The differences in envelope amplitude during pre-stimulus (–2.5 to –0.5 s) and post-stimulus (0–2 s) interval were compared by means of a random permutation cluster *t*-test with Monte Carlo estimates. To rule out the possibility that these differences drive the effects observed on the source-level, the envelope differences were correlated with the ERΔ_Pow_ values obtained earlier. For comparison, the same analysis was performed for the stimulation and sham group. For the sham group, envelope differences should reflect the event-related suppression of alpha-power, commonly observed during MR, and therefore highly correlate with the source-level ERΔ_Pow_. Pre- versus post-stimulus envelope differences in the stimulation group, however, should predominantly reflect changes in the tACS artifact. High correlations between sensor-space envelope differences and source-level ERΔ_Pow_ would thus indicate that systematic modulations of the tACS artifact drive changes in ERΔ_Pow_, rather than an actual physiological effect of tACS.

#### Experimental design and statistical analysis

Statistical analysis was realized in a 2 × 6 mixed-effects repeated-measures design with the between subject factor condition (stimulation versus sham) and the within subject factor block (6 levels). The normalized behavioral (performance, RTs) and physiological (ERΔ_Pow_) data were analyzed using repeated-measures ANOVAs (rmANOVA). Greenhouse–Geisser corrected *p*-values are reported where appropriate. If significant interactions between condition and block were revealed, analysis was subsequently split into two separate rmANOVAs, one covering the effects during stimulation (factors condition, stimulation vs. sham; block, block 2 vs. block 3) and the other analyzing outlasting effects (factors condition, stimulation vs. sham; block, block 4 vs. block 7). Comparisons of single blocks were performed using two-sample *t*-tests. Generalized η^2^ and Cohen’s *d* values are reported as measures of effect size. Pearson’s correlation coefficients were calculated to relate behavioral and physiological effects, as well as physiological effects and stimulation intensity.

Statistical analysis was performed using R 3.2.3 (The R Core Team, R Foundation for Statistical Computing). Cluster-based permutation tests on MEG data were performed in Matlab 2016a using statistical functions implemented in the Fieldtrip toolbox (Oostenveld et al., 2011).

#### Code accessibility

All scripts underlying the presented results are available as Extended Data and can be accessed online via the open science framework: https://osf.io/btnu7/.

10.1523/ENEURO.0069-18.2018.ed1 Extended Data Supplementary Matlab Code. Download Extended Data, ZIP file.

## Results

### Behavioral results

A Welch’s two-sample *t*-test yielded a trend for slightly better raw task performance in the baseline block for the sham group compared to the stimulation group (*t*_14.9_ = –2.00, *p* = 0.06, *d* = 0.9; *M_stim_* = 87.3%, *SD* = 3.6%; *M_Sham_* = 91.7%, *SD* = 5.9%). The rmANOVA on relative performance change revealed a significantly larger facilitation of MR performance, relative to baseline, in the stimulation group compared to sham (condition: *F_1,18_* = 4.93, *p* = 0.04, η^2^ = 0.14). Average performance during and after stimulation was *M_Stim_* = 92.3% (*SD* = 2.5%) and *M_Sham_* = 90.9% (*SD* = 5.6%), respectively.

**Figure 2.**
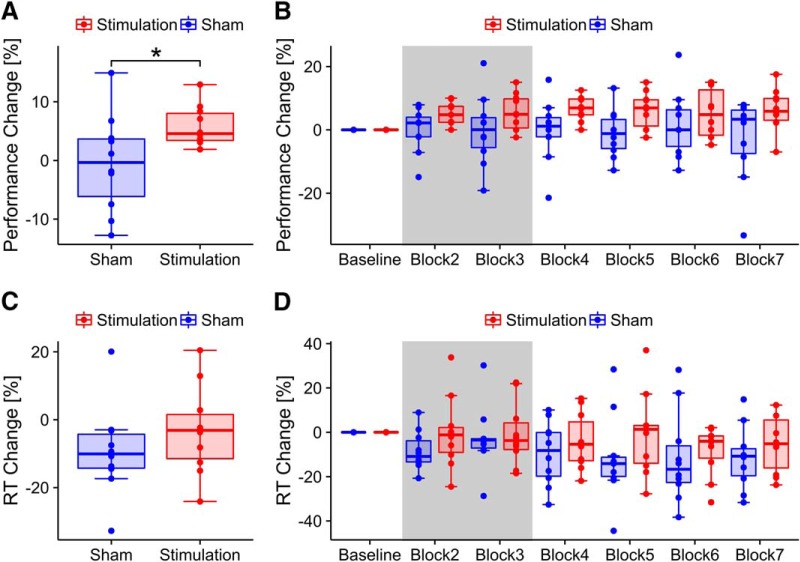
Behavioral results. ***A***, Change in task performance for stimulation and sham group, relative to baseline, pooled over all experimental blocks. Boxes indicate the 25th and 75th percentile of the sample distribution (interquartile length); lines inside the boxes mark the median. Whiskers extend to the most extreme values within 1.5 times the interquartile length. Asterisks code for significance (*, *p* < 0.05). ***B***, Change in task performance relative to baseline for stimulation and sham group depicted over experimental blocks. The gray area indicates blocks that were performed during tACS or sham stimulation. ***C***, Change in RT for stimulation and sham group relative to baseline pooled over experimental blocks. ***D***, Change in RT for stimulation and sham group relative to baseline depicted over experimental blocks. Gray area indicates blocks that were performed during tACS or sham stimulation.

Experimental groups did not differ with respect to their baseline RTs (*t*_16_ = 0.3, *p* = 0.77, *d* = 0.13, *M_Stim_* = 2763 ms, *SD* = 848 ms, *M_Sham_* = 2660 ms, *SD* = 659 ms). Analysis of the normalized RTs revealed a trend for the factor block (*F*_5,90_ = 2.47, *p* = 0.07, η^2^ = 0.03), but no effect of stimulation (*F*_1,18_ = 1.02, *p* = .33, η^2^ = 0.04). Mean reaction times during and after stimulation were *M_Stim_* = 2597 ms (*SD* = 710 ms) and *M_Sham_* = 2371 ms (*SD* = 524 ms) on average. Results of the behavioral analysis are summarized in Fig. 2.

### Event-related alpha modulation

**Figure 3.**
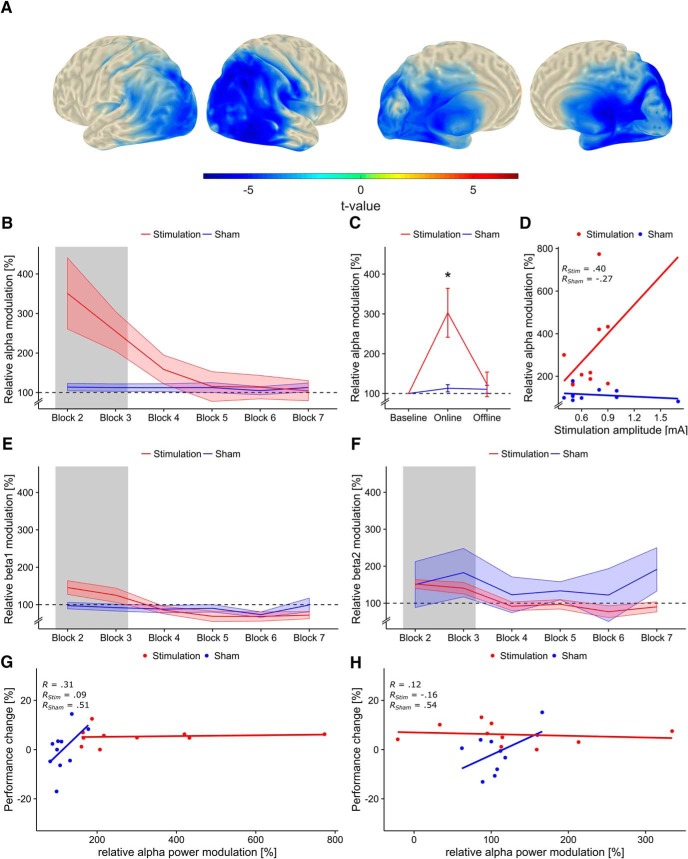
Event-related alpha-power modulation. ***A***, Region of interest (ROI). Significant cluster (pre- vs. post-stimulus power) in the IAF-band during the first block before tACS or sham stimulation, computed over the whole sample (*p_cluster_* < 0.001). Topographies depict *t*-values mapped on an MNI standard surface. Statistical maps are thresholded at α < 0.01. The depicted cluster (blue) was used as ROI to extract the time course of alpha-power modulation, relative to baseline, over blocks from the virtual channels. ***B***, Relative alpha-power modulation within ROI depicted for each block. The gray area indicates blocks during tACS or sham stimulation. Shaded areas represent standard error of the mean (SEM). Dashed line depicts baseline level. ***C***, Relative alpha-power modulation during tACS or sham (online) and after stimulation (offline). Error bars represent SEM; asterisks code for significant differences (*, *p* < 0.05). ***D***, Relative alpha-power modulation during stimulation correlated with stimulation intensity. Each point represents a single subject’s stimulation amplitude and relative alpha-power modulation, averaged over the two stimulation blocks (blocks 2 and 3). Please note that a stimulation intensity was determined for all participants (including sham); however, only participants in the stimulation group had this intensity continuously applied during blocks 2 and 3. ***E***, Relative power modulation in the lower beta-band (IAF + 3 Hz to IAF + 11 Hz) within the ROI for each block. ***F***, Relative power modulation in the higher beta-band (IAF + 12 Hz to IAF + 20 Hz) within the ROI for each block. ***G***, ***H***, Correlation between change in task performance and relative alpha-power modulation during (***G***) and after (***H***) tACS. High, albeit nonsignificant, correlations were evident for the sham group, but not the stimulation group.

**Figure 4.**
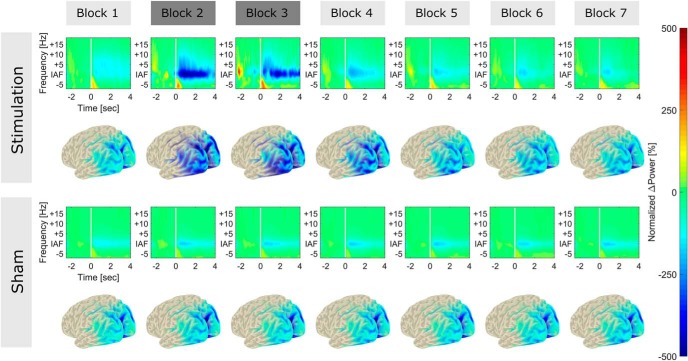
Normalized, baseline-subtracted TFRs and source topographies. TFRs and source topographies for stimulation (***top rows***) and sham group (***bottom rows***). TFRs were aligned at IAF and averaged over subjects in each group. The range from –2.5 to –0.5 before stimulus onset (white bar) served as reference period for baseline subtraction. Spectra were subsequently normalized by the power difference in the alpha-band (IAF ± 2 Hz) during the baseline block (block 1) before stimulation. Normalization was performed such that the data presented resemble data in the statistical analysis. Blocks 2 and 3 (dark gray) represent data acquired during tACS or sham stimulation. All other blocks (light gray) were measured in absence of stimulation. Functional maps were averaged over subjects and projected onto an MNI standard surface. Only activity within the analyzed ROI is depicted. A strong facilitation of event-related power modulation around the IAF can be observed during tACS application (block 2 and 3).

Comparison of pre- and post-stimulus IAF-band power, during the baseline block, revealed a significant cluster in occipito-parietal areas (*p_cluster_* < 0.001; Fig. 3*A*) for the whole sample. The identified cluster was used as an ROI to extract the time course of ERΔ_Pow_ from the different blocks and to limit the subsequent analysis to physiologically meaningful brain regions. The subsequent rmANOVA revealed a significant main effect of block (*F*_5,90_ = 7.22, *p* = 0.009, η^2^ = 0.15) as well as a significant condition*block interaction (*F*_5,90_ = 6.81, *p* = 0.011, η^2^ = 0.15), and a trend for the main effect of condition (*F*_1,18_ = 3.62, *p* = 0.07, η^2^ = 0.10). Please refer to Fig. 3*B* for an overview of the time course of relative ERΔ_Pow_. To further resolve the significant interaction, separate rmANOVAs were performed on the data acquired during and after tACS. These analyses exhibited a significant main effect of condition (*F*_1,18_ = 9.34, *p* = 0.007, η^2^ = .27) during stimulation, but not thereafter (condition: *F*_1,18_ = 0.14, *p* = 0.71, η^2^ < 0.01; Fig. 3*C*). Furthermore, a significant effect of block (*F*_3,54_ = 3.55, *p* = 0.02, η^2^ = 0.02), as well as a significant condition*block interaction (*F*_3,54_ = 3.10, *p* = 0.034, η^2^ = 0.02) were found in the post-stimulation data. None of the other main effects or interactions reached significance. It was not possible to further resolve the significant condition*block interaction during the post-stimulation blocks. Separately testing relative ERΔ_Pow_ values of the two experimental groups against each other did not reveal significant differences for any of the blocks (all p > 0.12, Welch two-sample *t*-test, one-tailed, uncorrected). Based on pure visual inspection, the interaction appears to be driven by a group difference during the first block after stimulation (block 4, see Fig. 3*B*), which might be indicative of a weak tACS aftereffect during this block. Refer to Fig. 4 for group-averaged time-frequency representations of participants’ normalized alpha-power change and the corresponding source-level topographies within the analyzed ROI.

No significant correlation between the increase in ERΔ_Pow_ during stimulation and stimulation intensity was observed in the stimulation group (*r* = 0.40, *t*_8_ = 1.25, *p* = 0.24). A weak, negative, non-significant correlation was observed in the sham group (*r* = –0.26, t_8_ = –0.78, *p* = 0.45; Fig. 3*D*).

To test whether the effects of tACS were specific to the alpha-band, the analysis was repeated on event-related power modulations in the lower (IAF + 3 Hz to IAF + 11 Hz) and upper (IAF + 12 Hz to IAF + 20 Hz) beta-bands within the ROI. The rmANOVA for the lower beta-band revealed a significant effect of block (*F*_5,90_ = 15.10, *p* < 0.001, η^2^ = 0.17) as well as a significant condition*block interaction (*F*_5,90_ = 9.37, *p* < 0.001, η^2^ = 0.11). Two separate rmANOVAs, testing the effects during and after stimulation, revealed a trend for the factor condition during stimulation (*F*_1,18_ = 4.17, *p* = 0.056, η^2^ = 0.18) as well as a significant effect of block *(F*_1,18_ = 4.72, *p* = 0.043, η^2^ = 0.02). After stimulation, only a trend for the factor block was found (*F*_3,54_ = 2.28, *p* = 0.09, η^2^ = 0.03). No significant effects were found in the analysis of the upper beta-band. Fig. 3*E*, *F* summarizes results for the lower and upper beta-band analysis (all *p* > 0.1).

There were no significant correlations between relative ERΔ_Pow_ and change in task performance during (*r_online_* = 0.3, *t*_18_ = 1.37, *p* = 0.18) or after (*r_offline_* = 0.11, *t*_18_ = 0.49, *p* = 0.62) stimulation. Descriptively, the correlation was higher for the sham group both during and after stimulation (*r_Sham/online_* = 0.51, *t*_8_ = 1.67, *p* = 0.13; *r_Sham/offline_* = 0.54, *t*_8_ = 1.83, *p* = 0.1) compared to the stimulation group (*r_Stim/online_* = 0.09, *t*_8_ = 0.27, *p* = 0.8; *r_Stim/offline_* = –0.16, *t*_8_ = –0.45, *p* = 0.67; Fig. 3*G*, *H*).

### Control analyses

**Figure 5.**
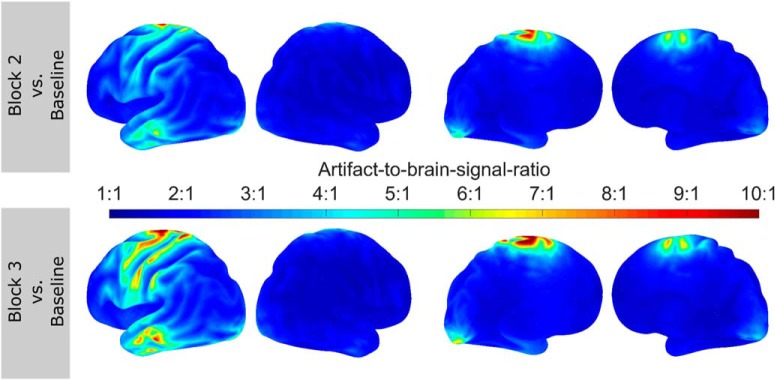
Artifact-to-brain-signal topographies. Topographies depict the average ratio between participants’ pre-stimulus alpha-power, estimated during the baseline block, and residual artifact in the pre-stimulus interval during block 2 (***top row***) and 3 (***bottom row***). Results are depicted only for the stimulation group. The ratio is strongest in central areas covered by the stimulation electrodes and cables. Frontal and posterior areas within the ROI seem less affected, with the ratio falling in a physiologically plausible range (<1:4), such that residual artifact and facilitatory effects of the stimulation or spontaneous increase of alpha power cannot be disentangled. Results have to be interpreted in terms of an upper boundary for the size of the residual artifact, as each virtual channel contains a mixture of brain signal of interest and artifact.

To rule out the possibility that the strikingly strong facilitation of power modulation in the alpha-band was driven by residual artifacts, several control analyses were performed. In a first step, the performance of the artifact suppression achieved by LCMV was evaluated. To this end, the ratio of pre-stimulus alpha-power during the (tACS-free) baseline block and the two tACS blocks was compared in sensor- and source-space. On average, this artifact-to-brain-signal ratio was 2,534,000:1 in block 2 and 2,569,000:1 in block 3 (average over all sensors and subjects) in the sensor-space data. After LCMV beamforming, the ratio was reduced to 2.72:1 in block 2 and 3.13:1 in block 3 (average over virtual sensors and subjects). The largest ratio observed in a single virtual channel of one subject after beamforming was 93.42:1. Fig. 5 illustrates the spatial distribution of the artifact-to-brain-signal ratio on the source-level. The ratio was highest in central areas, covered by stimulation electrodes and cables. Outside of these areas, the ratio was substantially smaller and falls within a physiologically plausible range for alpha-band oscillations (<4:1). Overall artifact suppression appeared to be slightly worse during block 3 compared to block 2.

**Figure 6.**
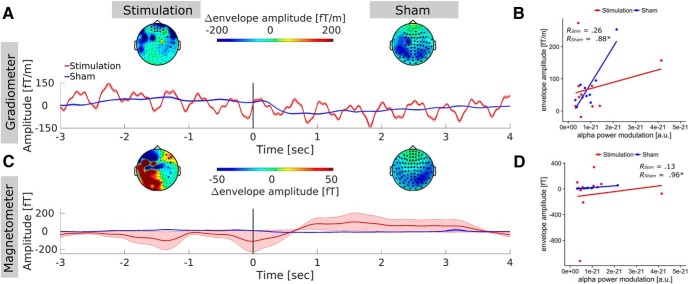
Event-related artifact envelope. ***A***, Topography and time course of the artifact envelope around stimulus onset in gradiometer sensors. Topographies represent the amplitude difference of the envelope, around the stimulation frequency between the reference (–2.5 to –0.5 s) and the testing periods (0–2 s). Darkened sensors mark locations in which this difference was significant. Data of the sham group is depicted for comparison and reflects the task-related modulation of endogenous alpha oscillations (visible shortly after stimulus onset, vertical black bar at 0 s) as no stimulation artifact was introduced to the data. Envelope epochs of all subjects were demeaned before averaging to enhance comparability of the envelope modulation. Shaded areas depict standard error of the mean (SEM). Gradiometer time courses were strongly dominated by rhythmic modulation around 1–2 Hz that potentially reflects a technical artifact in this sensor type. ***B***, Correlation between event-related modulation of the artifact envelope in gradiometer sensors and event-related alpha-power modulation within the ROI after beamforming. The absence of a significant (or even moderately high) correlation in the stimulation group provides supporting evidence that the effects observed in source-space are not driven by systematic event-related modulations of tACS artifact strength. ***C***, Topography and time course of the artifact envelope around stimulus onset in magnetometer sensors. ***D***, Correlation between event-related modulation of the artifact envelope in magnetometers and alpha-power modulation within ROI after beamforming. Similar to the gradiometer data, no correlation between source-level effects and artifact tACS artifact modulation was observed.

The event-related envelope of the sham group was consistent with the pattern of alpha-power decrease typically observed after stimulus onset in the MR task in both sensor types. This was confirmed by the permutation cluster analysis, which revealed significant positive clusters in the magnetometer and the gradiometer data (*p_cluste_*_r_ < 0.001, Fig. 6*A*, *C*; significant sensors are marked by black dots), and further supported by the high correlation between source-level power modulation and envelope difference of magnetometer (*r* = 0.96, *t*_8_ = 10.17, *p* < 0.001; Fig. 6*B*) and gradiometer (*r* = 0.88, *t*_8_ = 5.23, *p* < 0.001; Fig. 6*D*) channels. In the stimulation group, time course and topography of the envelope overall exhibited the opposite pattern, with lower amplitudes before stimulus onset and increased amplitude thereafter. In addition, the envelope time course of gradiometers shows a prominent rhythmic activity in the range of 1–2 Hz. This could potentially reflect heartbeat-related modulations of the tACS waveform (Noury et al., 2016). However, given that this rhythmic activity was observed in only one sensor type and in a relatively systematic manner, it more likely reflects a technical artifact. Importantly, no such rhythmic modulation was evident in the time–frequency representations after LCMV (Fig. 4). Results of the cluster analysis revealed positive clusters in the gradiometer data in only a few frontal sensors *(p_cluster_* < 0.05; Fig. 6, top left) as well as positive and negative clusters for some magnetometer channels (*p_cluster_* < 0.05). No significant correlation was evident between the observed source-level power modulations and the sensor-level envelope differences in magnetometer (*r* = 0.13, *t*_8_ = 0.37, *p* = 0.72) or gradiometer sensors (*r* = 0.26, *t*_8_ = 0.75, *p* = 0.47). Overall, results do not support the idea that the effects observed on the source-level can be explained by systematic, task-related changes in artifact strength. Very few channels were found to exhibit significant, task-related power modulations. Those that did rather seemed so show a reversed pattern of artifact modulation compared to the source-level data.

## Discussion

To date, few studies have investigated the effects of tACS on oscillatory activity in the human brain during stimulation (Helfrich et al., 2014; Voss et al., 2014; Ruhnau et al., 2016), due to the massive electromagnetic artifact encountered during the measurement. The current study adds to this line of research by characterizing how event-related oscillatory activity during a cognitive task reacts to externally applied perturbations in the same frequency band. Theoretically, tACS could counteract, overwrite, or enhance the oscillations underlying performance of the task.

Results show that, rather than counteracting or overwriting the event-related down-regulation of oscillatory power during the mental rotation (MR) task, continuous application of tACS facilitated the pre-existing difference between pre- and post-stimulus power in the alpha-band. This finding indicates that tACS exerts its effects differently during pre- and post-stimulus intervals. Given that tACS is usually observed to facilitate power of the targeted brain oscillation after stimulation, the current finding seems most likely to be caused by stronger enhancement of alpha-power before stimulus onset (Neuling et al., 2013; Veniero et al., 2015; Kasten and Herrmann, 2017), rather than inhibition of post-stimulus alpha-power. Unfortunately, this cannot be resolved using the current data, as the contrast between pre- and post-stimulus intervals was necessary to account for residual tACS artifacts. To directly observe differential effects of tACS on event-related brain oscillations, future work might make use of amplitude-modulated tACS (AM-tACS), which has been proposed as a strategy to overcome the strong electrophysiological artifact in the range of the targeted brain oscillation (Witkowski et al., 2016). This new stimulation waveform has very recently been shown in a computational model to exhibit entrainment mechanisms similar to those of conventional sine-wave tACS (Negahbani et al., 2018). However, it should be noted that two recent studies cast doubts on whether AM-tACS is entirely free of stimulation artifacts in the range of the targeted brain oscillation (Minami and Amano, 2017; Kasten et al., 2018). Thus, careful assessment of brain signals recorded during stimulation would still be required.

A differential effect of tACS on pre- and post-stimulus intervals can be interpreted in terms of a short-scale state dependence of tACS effects. Several studies have demonstrated that tACS effects are state-dependent on larger time scales. On the one hand, tACS in the alpha-band seems to only be effective when the targeted brain oscillation is comparatively low in amplitude, e.g. during eyes open, but not during eyes closed (Neuling et al., 2013; Alagapan et al., 2016; Ruhnau et al., 2016). On the other hand, involvement of the targeted brain oscillation in a given state (or task) also seems necessary to successfully induce tACS effects (Feurra et al., 2013). In the simplest case, pre- and post-stimulus intervals in the current study reflect two distinct brain states (a resting or preparatory state and an MR state) that differ in terms of alpha-oscillation involvement and susceptibility to tACS. This pattern is in line with predictions derived from synchronization theory, which require the presence of a self-sustained oscillator for entrainment to occur (Pikovsky et al., 2003). Consequently, tACS might exhibit its effect during the pre- but not during the post-stimulus interval where alpha oscillations are suppressed due to the task.

Although the current findings converge with observations of facilitated event-related desynchronization (ERD) after tACS (Kasten and Herrmann, 2017), it is important to emphasize that online effects of tACS (during stimulation) cannot directly be inferred from effects measured after stimulation. While computational models and animal experiments suggest entrainment as the core mechanism of online tACS effects (Fröhlich and McCormick, 2010; Ozen et al., 2010; Reato et al., 2010), there is increasing evidence that the aftereffects of tACS might be better explained by mechanisms of neural plasticity (Zaehle et al., 2010; Vossen et al., 2015). Different mechanisms of action, during and after stimulation, could in principle lead to different effects of tACS on event-related oscillations. Thus, direct observations of tACS online effects are inevitable to predict and understand behavioral outcomes of tACS experiments.

The observed enhancement of event-related alpha-power modulation can explain previous results of better performance in the MR task during tACS (Kasten and Herrmann, 2017). Mental rotation tasks typically feature alpha oscillations before stimulus onset, followed by task-induced suppression of the oscillation. The suppression typically lasts until participants finish task execution (Michel et al., 1994). Studies using repetitive transcranial magnetic stimulation (rTMS) and neurofeedback training (NFT) have demonstrated facilitated MR performance when targeting spontaneous alpha oscillations during the pre-stimulus interval (Klimesch et al., 2003; Hanslmayr et al., 2005; Zoefel et al., 2011). More broadly, alpha oscillations have been suggested to enhance performance, in a variety of tasks, by suppressing activity in task-irrelevant areas of the brain or in preparation for an upcoming event, which has been referred to as “gating by inhibition” (Jensen and Mazaheri, 2010). By selectively enhancing prestimulus alpha-power, tACS could facilitate the preparatory gating and thus benefit subsequent task performance.

While the results are in agreement with previous findings (Kasten and Herrmann, 2017), they contradict observations of Neuling et al. (2015). That study reported a tendency for reduced alpha desynchronization elicited by a passive visual task during tACS. However, the authors calculated relative change (computed similarly to ERD/ERS) to capture event-related alpha desynchronization, which is vulnerable to residual artifacts in the data. As shown in Eqs. 2 and 3, such a residual artifact would lead to a biased (larger) denominator, resulting in systematic underestimations of ERD within the stimulated frequency band. Using the absolute power difference (here termed ERΔ_Pow_) between two time intervals within the same stimulation condition (i.e., pre-/post-stimulus alpha-power) appears to be a more robust measure to capture online effects of tACS. Using such a procedure, the residual artifact cancels out during the subtraction process. Importantly, this cancelation assumes that the strength of the residual artifact is relatively stable between conditions and uncorrelated with the task. Such systematic modulations could in principle occur if the task elicits systematic changes in physiological processes like heartbeat, respiration, or skin conductance (Noury et al., 2016). While there was no evidence for such a systematic change in artifact strength that could explain the observed pattern in the current data, the possibility has to be taken into account when using stimuli that can elicit stronger physiological responses (e.g., emotional pictures or demanding motor tasks). However, the impact of these modulations on the artifact suppression, compared to the size of the physiological effect on the brain, has not been thoroughly characterized yet.

In addition to the observed effect of tACS on power modulations in the alpha-band, the data revealed a trend toward increased event-related power modulations in the lower beta-band during tACS. This observation could be indicative of a rather unspecific effect of tACS (Kleinert et al., 2017). Alternatively, the effect in the lower beta-band could be explained by entrainment or as a resonance phenomenon at the first harmonic of subjects’ stimulation frequency (Herrmann, 2001; Herrmann et al., 2016a). Further, cross-frequency interactions between alpha and beta oscillations (Palva et al., 2005) could underlie the effects, resulting in co-modulation of beta oscillations stemming from tACS effects in the alpha-band.

Contradicting the previous finding of a prolonged, tACS-induced ERD increase in the alpha-band (Kasten and Herrmann, 2017) and despite the substantial online effects, only a short-lasting aftereffect during the first block after stimulation was observed, if at all. Several studies have successfully shown persistent effects of tACS on alpha-power during rest (Neuling et al., 2013; Veniero et al., 2015; Vossen et al., 2015; Kasten et al., 2016). A possible explanation for the lack of a sustained tACS effect in the current study was the relatively low stimulation intensity compared to the aforementioned experiments.

Similar to previous work (Kasten and Herrmann, 2017), a significantly stronger increase in MR performance was observed in the stimulation group compared to the sham group. Unfortunately, it cannot be ruled out that this effect might have been partly driven by differences in baseline performance between the two groups. This could also explain the absence of previously observed correlations between performance increase and facilitated alpha-power modulation (Kasten and Herrmann, 2017), which would have further supported the physiological findings. Alternatively, the strong effect of tACS on participants’ alpha-power modulation during stimulation might have caused ceiling effects such that, beyond a certain level, MR performance could not be facilitated any further. However, due to the differences in baseline performance, interpretability of the current behavioral results is limited. Nonetheless, this does not contradict the physiologic effects, which were the main focus of the current study. MR tasks induce comparably long-lasting event-related power modulations (Michel et al., 1994), a beneficial property when studying tACS effects on event-related oscillations. In the current experiment, this came at the cost of overall high task performance in both groups. Future studies might therefore benefit from more difficult MR paradigms (e.g., only including large rotation angles).

In addition to investigating the concurrent effects of tACS on event-related oscillations, the current study made an attempt to quantify the artifact suppression capabilities of LCMV beamforming. To this end, the oscillatory power around the stimulated frequency during tACS was compared to an artifact-free estimate of participants’ natural brain signal (alpha-power). This allowed to estimate the magnitude of the stimulation artifact relative to the brain signal of interest before and after artifact suppression. In the current study, this artifact-to-brain-signal-ratio was reduced from >2,500,000:1, before LCMV, to ∼3:1 thereafter, with stronger artifacts around stimulation electrodes and cables (∼10:1). Since the power values obtained during stimulation will always contain a mixture of residual tACS artifact and brain signal, this ratio can provide only an upper boundary for the size of the residual artifact. Alpha-power increase, by a factor of 3 or 4, falls into a physiologically plausible range for spontaneous of stimulation-induced alpha-power changes, consistent with previous work on tACS aftereffects (Neuling et al., 2013; Kasten and Herrmann, 2017; Stecher et al., 2017). The artifact-to-brain-signal ratio might nevertheless be a useful tool for future studies to assess whether a residual artifact falls within the same order of magnitude as the brain signal of interest. It might also be used to evaluate and optimize the performance of artifact suppression techniques, i.e., by tuning relevant parameters. Thus far, artifact suppression approaches have mostly been evaluated subjectively, i.e., by inspecting raw time series (time-) frequency spectra or ERPs (Helfrich et al., 2014; Neuling et al., 2015; Witkowski et al., 2016). The artifact-to-brain-signal ratio provides a more objective evaluation of the artifact size, relative to the brain signal of interest, and is scale-free, allowing for easy comparison of different artifact suppression approaches even between different measurement modalities (EEG/MEG, LCMV, template subtraction, etc.). In addition, the mapping of residual artifact strength allows the assessment of overlap between hot spots of residual artifacts and regions of interest.

The findings presented in the current study provide the first direct insights concerning the online effects of tACS on event-related oscillations in humans. The effects were investigated using a rather simplistic approach, using only two conditions (stimulation vs. sham) and one stimulation frequency, targeting posterior alpha oscillations with a Cz-Oz montage. This path was chosen to establish an analysis framework, including controls, for the investigation of concurrent effects of tACS. Success at this stage would greatly facilitate approaches with more complex designs requiring larger sample sizes and higher computational efforts. TACS experiments generally allow for a multitude of control and contrast conditions, including alternative electrode montages and frequencies. The current study can therefore neither resolve frequency nor montage specificity of tACS effects. However, with the present results and the proposed analysis pipeline, the current study paves the way for further investigations of montage and frequency specificity of tACS effects, specifically on event-related oscillatory dynamics during various cognitive tasks.

## Synthesis

Reviewing Editor: Satu Palva, University of Helsinki Decisions are customarily a result of the Reviewing Editor and the peer reviewers coming together and discussing their recommendations until a consensus is reached. When revisions are invited, a fact-based synthesis statement explaining their decision and outlining what is needed to prepare a revision will be listed below. The following reviewer(s) agreed to reveal their identity: J. Palva. Note: If this manuscript was transferred from JNeurosci and a decision was made to accept the manuscript without peer review, a brief statement to this effect will instead be what is listed below.

Synthesis:

Both reviewers found your manuscript addressing a methodological challenge that is fundamentally important for obtaining understanding of the nature and locations of brain processes that tACS modulates. The paper was found to be well written, analyses methods were found to be sound, and careful supplemental analyses were found to be conducted to rule out that the results can be merely attributed to residual artifacts. Nevertheless, both reviewers had some suggestions on how to improve the manuscript. Also, as additional aspects the reviewers concluded that it would be important if the novel parts of the program code could be released with the manuscript for the community to have access for the new method.

Reviewer #1

Kasten et al. used concurrent tACS-MEG to investigate the effects of tACS (vs. sham) to the visual cortex (Oz-Cz montage) at individual alpha frequency during a mental rotation task on both behavioral performance and the event-related alpha desynchronization associated with the task. They report tACS to increase both mental rotation performance and event-related alpha (and beta) desynchronization in response to the onset of the (to be rotated) visual stimulus.

I have reviewed an earlier submission of this manuscript to another journal before and will therefore merely asses to what degree my pervious concerns have been addressed.

The paper is well written, analyses methods are sound, and careful supplemental analyses have been conducted to rule out that the results can be merely attributed to residual artifacts. In particular, an effective subtraction approach of different task periods with the same stimulation artifacts warrants sufficient artifact removal. Additionally, the calculation of an artifact-to-brain-signal-ratio provides an interesting new index to assess the artifact topographies and the efficacy of artifact cleaning procedures. The authors have addressed all of my previous concerns to the extent possible without conducing further experiments. They have toned down their conclusions a level that is supported by the data, and they do sufficiently discuss the shortcomings of the study.

While no control frequency or montage have been used, the authors now take these limitations into account. They also analyzed neighboring frequency bands, such as the beta band (and found effects in that band). Further, the choice of a between-subject design (instead of a within-subject design) introduces additional variance, allows for imbalanced confounding factors (like gender here), and prevents appropriate correlations between tACS effects (stim-sham) on oscillatory activity and behavior. However, the authors discuss these aspects and other aspects sufficiently and give directions for future studies. I am still not entirely sure what the results tell us about the function of alpha oscillations in mental rotation or how exactly alpha-tACS may impact their event-related desynchronization, and I encourage the authors to elaborate a bit more on these points. What would be more relevant for high mental rotation performance: a low amplitude of alpha oscillations in absolute terms (supposed to reflect or even mediate increased excitability of the cortex) or merely a strong reduction relative to the pre-stimulus level, even if it may result from higher pre-stimulus levels rather than lower levels during mental rotation? And given that the subtraction of these two periods is needed for tACS-artifact removal, how can this question be assessed in the current study?

Despite the existing (and discussed) shortcomings, the presented study nonetheless reports very interesting data and provides important perspectives on tACS-MEG artifact cleaning: I thus support the publication of this manuscript.

Remaining minor points

1. Line 153: Which sensors have been selected? Maybe indicate in Figure 1B.

2. Line 242: “Epochs were then projected into sensor space using the previously obtained beamformer filters, [...]” Shouldn't it be ‘source space’ instead of ‘sensor space’ here?

3. Line 380: “A non-significant correlation was observed [...] p = .24 [...]” should be “No significant correlation was observed [...]”. What is the sense of doing inferential statistics otherwise?

Reviewer # 2

This study addresses methodological challenge of acquiring electrophysiological measurements, here MEG, during tACS stimulation. This is fundamentally important for obtaining understanding of the nature and locations of brain processes that tACS modulates. Furthermore, the authors show that in a mental rotation task, tACS enhances the induced post-stimulus amplitude modulation (suppression) of alpha-band oscillations. This is a very important result showing non-invasively a direct modulation of brain dynamics by tACS. Regardless of its shortcomings, the manuscript clearly advances the field and merits publication.

Comments

As far as I see, the statistical analyses have been performed appropriately and rigorously, although the N is low. The authors should take special precautions against false positive findings given that only 10 subject are compared against other 10.

I would hesitate advertising the MR performance effect in the visual abstract as it is of dubious reliability because of the near-significant baseline effect and very low N. The authors could consider whether the current data could be used here in conjunction with previous data published in Kasten and Herrmann, 2017, it the dataset there is different (?). I.e., to treat the present behavioral data as a replication cohort for the previous and assessing the quantitatively the replicability of the result. In the illustration of the behavioral data (Fig. 2), the authors could consider more modern methods for data illustration than the ‘currently infamous’ bar plots so that also the individual values and distributedness could be evaluated. For the behavioral data, please also report the reaction times, not just changes.

Analyses are done in Matlab but, as far as I see, the software is not released and is hence untestable. The authors advertise establishing a new ‘pipeline’ with the proposed procedure: it would be crucial for the community to have an access to this pipeline also in terms of program code.

The physiological/functional implications of alpha ERD enhancement could be discussed in more detail. Would the apparent lack of correlation between performance improvement and tACS-alpha-ERD enhancement (Fig. 3G) suggest that alpha ERD is inconsequential for task performance?

The manuscript makes a core (perhaps implicit?) assumption that the effects of alpha tACS on alpha ERD arise directly from an interaction of these two alpha processes, and consequently the effect of alpha tACS on beta-band amplitudes results from beta being a harmonic of alpha. Let me propose an alternative hypothesis: the effects of tACS influence the cortical state so that the primary effects of the stimuli are greater, which results in greater induced alpha ERD afterwards. ‘Primary effects’ here mean the evoked response and the corresponding transient delta/theta-amplitude increase. Would the present data rule out this hypothesis? Long-duration of the stimuli probably preclude it as an alternative explanation but on the other hand, the data in Fig. 4 are suggestive of an early delta/theta amplitude effect as well.
